# Are unmet health related social needs associated with emergency department utilization among Medicare beneficiaries?

**DOI:** 10.1186/s12913-025-12554-7

**Published:** 2025-03-31

**Authors:** Ethan E. Abbott, Shameeke Taylor, Carmen Vargas-Torres, Kevin Petrozzo, David G. Buckler, Lynne D. Richardson, Alexis M. Zebrowski

**Affiliations:** 1 Department of Emergency Medicine, Icahn School of Medicine at Mount Sinai, 1 Gustave L. Levy Place, Box 1620, New York, NY 10029 USA; 2 Department of Population Health Science and Policy, Icahn School of Medicine at Mount Sinai, New York, NY USA; 3 Institute for Health Equity Research (IHER), Icahn School of Medicine at Mount Sinai, New York, NY USA; 4 Division of Data-Driven and Digital Medicine, Icahn School of Medicine at Mount Sinai, New York, NY USA

**Keywords:** Medicare current beneficiary survey, Health-related social needs, Emergency department

## Abstract

**Background:**

Health-related social needs (HRSN) are increasingly recognized as important factors influencing healthcare outcomes and utilization. This study examined the association between unmet HRSNs and emergency department (ED) utilization among Medicare beneficiaries.

**Methods:**

We conducted a retrospective, survey-weighted cohort analysis of the 2015–2016 Medicare Current Beneficiary Survey (MCBS) linked with Medicare fee-for-service claims. The study included beneficiaries aged ≥ 65 years enrolled in fee-for-service Medicare who completed the MCBS. The primary predictor was having ≥ 1 unmet HRSN (food insecurity, delaying care due to cost, or difficulty accessing medical care). Primary outcomes included an index ED visit (1 ED visit) and any ED revisit within one year (≥ 2 ED visits); hospital admission from these ED visits was a secondary outcome. We fit multivariable logistic regression models adjusted for demographic, socioeconomic, and clinical factors. Interactions were tested using adjusted Wald tests.

**Results:**

Among 16,990 beneficiaries, 6.2% (*n* = 1,046) reported one or more unmet HRSNs. Within one year of completion of the survey, 27.7% (*n* = 4,702) had an 1 ED visit, with 9.03% of all beneficiaries (*n* = 1,535) requiring admission to the hospital. In adjusted analyses, beneficiaries with unmet HRSN had significantly higher odds of ≥ 2 ED visits (OR 1.47, 95% CI 1.12–1.91) compared to those without unmet HRSNs, but not for index ED visit. The oldest age category (85 + years) showed significantly increased odds of both index ED visits and revisits. Unmet HRSN were not significantly associated with risk of subsequent hospital admission for both index ED visit and ED revisit.

**Conclusion:**

Self-reported unmet HRSNs were associated with significantly increased odds of ≥ 2 ED visits but not an 1 ED visit within one year of the MCBS survey. These findings highlight the importance of improved and standardized data collection of HRSNs to understand the impacts on ED utilization. Oldest age patients had increased odds of index ED visits and revisits. Further investigation should focus on strategies to reduce ED recidivism in vulnerable older populations.

**Supplementary Information:**

The online version contains supplementary material available at 10.1186/s12913-025-12554-7.

## Introduction

Health-related social needs (HRSN) are important individual level non-clinical factors that are tied to healthcare outcomes and utilization [1]. These include domains such as food insecurity, housing instability, and financial hardship. Food insecurity, a well-researched issue, has been linked to various health outcomes, including metabolic syndrome, an increased risk of bone fractures in children, end-stage renal disease in adults, and higher mortality rates among immunocompromised patients and infants [2, 3]. Housing instability has also been associated with several negative health effects. In a large European study and was linked to a decline in self-reported health [4]. Similarly, in multiple US states, housing instability correlated with higher hospital visits for mental health issues and preventable conditions [5]. Financial hardship impacts numerous health-related factors, such as healthcare utilization and access to preventive care like vaccinations [6]. The wide range of adverse outcomes tied to HRSN underscores the significant role these factors play in individual level health outcomes.

HRSN are increasingly recognized as important data to collect for health systems, however this data has historically been less rigorously collected in a structured format or integrated into clinical practice at the individual level [7, 8]. This is despite growing evidence of how social needs can intersect with healthcare utilization and outcomes. Understanding the patterns of individual level unmet HRSN and their association with healthcare utilization is critical. This can inform the design of future targeted interventions while improving the care of vulnerable patients and addressing racial, ethnic, and socioeconomic disparities. Pertinent to our study, we are guided by the conceptual framework proposed by the World Health Organization and the United States Department of Health and Human Services Healthy People 2030, acknowledging the deep connection of HRSN with clinical outcomes [9]. This framework recognizes material and psychosocial conditions and health system factors—directly influence health outcomes and healthcare utilization patterns. This framework provides a structure for understanding how unmet social needs among Medicare beneficiaries may impact emergency department utilization through both direct and indirect pathways and informs our analytical approach to examining these relationships.

The Medicare Current Beneficiary Survey (MCBS) provides valuable information on several key HRSN domains for the Medicare population. Prior research has highlighted the high prevalence of unmet HRSNs among Medicare beneficiaries and their association with increased healthcare utilization, particularly in acute care settings such as the emergency department (ED) [10, 11]. Previous studies in the areas of ophthalmology, end of life care, and depression diagnoses have demonstrated unmet HRSN are associated with disparities in access to care, differences in adherence to treatment, and poor clinical outcomes among Medicare beneficiaries [11–13]. 

Identifying patterns of emergency department (ED) utilization for Medicare beneficiaries with unmet HRSN is critical to ensuring comprehensive and equitable care. Older adult beneficiaries with a high burden of unmet HRSN seen in the ED are likely to face unique healthcare challenges that impact care utilization and clinical outcomes. The challenges that come with aging may include decreased ability to perform activities of daily living due to mobility issues, poor eyesight, polypharmacy, comorbidity burden and cognitive impairments [14, 15], which can make it difficult for older adults to manage their HRSN and maintain good health. However, there is limited knowledge regarding the relationship between HRSN and healthcare utilization (ED visits and inpatient care) among older adults. More broadly, our aim was to better understand the impact of social factors are driving ED utilization that might be preventable or better managed through targeted social interventions rather than acute medical care. This understanding could inform more effective and appropriate resource allocation and care coordination strategies for Medicare beneficiaries.

To build on current research in this area, our study sought to further examine the relationship between unmet HRSNs, ED utilization, and subsequent hospital admissions among Medicare beneficiaries, an area not deeply explored among this population. Specifically, we sought to (1) assess the prevalence of individual HRSNs among age-eligible beneficiaries using the MCBS data, (2) examine if the presence of unmet HRSN was associated with differences in ED utilization and subsequent risk of hospital admission and (3) evaluate the association between ED and inpatient utilization with demographic factors and comorbidity burden in this Medicare population.

## Methods

### Study design

We performed a retrospective, survey-weighted, cohort analysis of the 2015–2016 MCBS dataset. We included adults aged 65 years or older, who were enrolled in fee-for-service Medicare and completed the MCBS during the study period. We focused exclusively on beneficiaries aged 65 and older as those under 65 qualify for Medicare through disability or end-stage renal disease and represent fundamentally different healthcare needs and utilization patterns. The MCBS is a continuous survey of a nationally representative sample of the Medicare population that uses a rotating panel design where beneficiaries are interviewed up to three times a year over a four-year period. The survey provides comprehensive data on beneficiaries’ health status, healthcare utilization, and expenditures. Our study utilized 2015–2016 MCBS data due to specific constraints of our data use agreement and funding to obtain this data through CMS. We had full data access to this two-year period despite the survey’s four-year panel design. We linked the MCBS files with Medicare fee-for-service inpatient and outpatient claims files for the same years to identify ED utilization and hospital admissions. We included claims up to 365 days from the date of the initial MCBS survey.

#### Primary predictor and study variables

For the primary predictor, we utilized three HRSN-specific MCBS questions regarding (1) food insecurity; (2) delays in medical care due to cost; (3) access to medical care (see Table S1 for specific MCBS survey questions). These three questions were selected based on literature review of the most commonly experienced HRSNs, MCBS data availability and expert opinion within the research team [16–18]. To model our clinical outcomes, a dichotomous social risk measure was created that included the absence or presence of ≥ 1 of the above 3 unmet HRSN. If a beneficiary identified as having HRSN ≥ 1 they were classified as meeting criteria. For our models, we included we included patient age category (65–74, 75–84, 85 + years), sex, race (White, Black, Other), ethnicity (Hispanic, Non-Hispanic, Unknown), comorbidities (derived from Master Beneficiary Summary File (MBSF) chronic condition segment file and categorized as 0–1, 2–3, or 3 + conditions), individual level income (≤$14,999, $15,000-$39,999, >$40,000), income-to-poverty ratio (≤ 100%, > 100–125%, > 125–150%, > 150–200%, > 200% of Federal Poverty Level), education level (High School graduate, Some College, College Degree, Unknown). Unless noted, the data for these covariates were obtained from the MCBS file.

### Outcomes

The primary outcome was ED utilization in the period up to 365 days following the initial MCBS survey date. We selected this outcome based on prior limited research examining MCBS survey respondents and ED utilization [19, 20]. We defined our outcome as the first ED visit (1 ED visit) and any subsequent ED revisit (≥ 2 ED visits) as ED utilization. To ascertain these outcomes, we utilized standardized methodology [21] using CMS inpatient and outpatient revenue files and filtering for ED visit revenue codes (0450–0459, 0981). We identified any emergency room charge amount > $0 and created a flag for claims that had ED-based charges. We merged this data with the MCBS beneficiary ID to ascertain our outcomes. Our secondary outcome was any hospital admission from the index ED visit and ED revisit. For this outcome, we also utilized the inpatient revenue file to ascertain both the admission source and matching revenue center codes (0450–0459) indicating emergency department services were utilized. We excluded elective admissions and transfers from other facilities to focus specifically on admissions originating from the ED.

### Statistical analysis

We used survey-weighted descriptive statistics to characterize the study population. Chi-square tests were used to compare characteristics between those with and without unmet HRSNs. We tested our associations using both unadjusted and fully adjusted models. For our fully adjusted models, we used multivariable logistic regression to quantify the relationship between unmet HRSN and ED utilization while controlling for sociodemographic (age, sex, race/ethnicity, income, education), and clinical (comorbidities) factors. From these models, we calculated odds ratios (ORs) and 95% confidence intervals (CIs) to estimate the association between unmet HRSNs and our outcomes of interest. We also conducted a separate analysis to examine for effect modification. We first fit a basic model examining the association between HRSN and each outcome, adjusted for covariates. We then tested interactions between HRSN and all covariates using adjusted Wald tests in survey-weighted logistic regression models. We tested interactions HRSN and all key covariates (age category, sex, race/ethnicity, chronic conditions, education, and income). All interaction models were adjusted for the same covariates as our main effects models.

All analyses were conducted using survey weights to account for the complex survey design of the MCBS. We utilized survey weights calculated by CMS (EVRWGTS15 and EVRWGTS16 files) to ensure that our study design included a nationally representative sample of Medicare beneficiaries. Data preparation and initial descriptive analyses were performed using R version 4.0.3 (R Foundation for Statistical Computing, Vienna, Austria). Complex survey analyses and multivariable logistic regression models were conducted using Stata version 16.1 (StataCorp, College Station, TX, USA). This study was completed in accordance with the STROBE guidelines [22]. This study was conducted in strict accordance with the ethical principles outlined in the Declaration of Helsinki. The research protocol was reviewed and approved by the Icahn School of Medicine at Mount Sinai Institutional Review Board. As the study utilized observational and retrospective data, the requirement for informed consent was waived by the approving committee.

## Results

### Overall

We identified a total of 16,990 beneficiaries who participated in the MCBS during the study period (Table 1). 42.7% (*n* = 7,260) of the cohort were female beneficiaries with a mean age of 78.6 years (SD 8.2). By race and ethnicity, 79.1% (*n* = 13,439) of beneficiaries were White, 8.46% (*n* = 1,438) were Black, and 9.26% (*n* = 1,574) were Hispanic. The income distribution revealed that 18.25% (*n* = 3,101) of beneficiaries had annual incomes of $14,999 or less, while 43.94% (*n* = 7,466) earned over $40,000. Educational attainment was relatively high, with 77.20% (*n* = 13,110) having some college education or a college degree. 57.70% (*n* = 9,798) of the cohort had incomes above 200% of the Federal Poverty Level. Overall, 6.20% (*n* = 1,046) reported ≥ 1 unmet HRSN. This included 1.07% (*n* = 181) with food insecurity, 3.30% (*n* = 560) with delaying medical care due to cost, and difficulty with accessing medical care 2.82% (*n* = 479). Examining overlap with unmet HRSNs, among the 181 Medicare beneficiaries who reported food insecurity, 39 beneficiaries also reported delaying healthcare due to financial concerns, and 17 beneficiaries also reported trouble accessing healthcare. During the study period 27.70% (*n* = 4,702) of all beneficiaries had an 1 ED visit within one year of the survey, with 9.03% (*n* = 1,535) of these visits resulting in admission. 12.90% (*n* = 2,195) of all beneficiaries had ≥ 2 ED visits with 6.40% (*n* = 1,081) resulting in a hospital admission.

**Table 1:** Overall study cohort characteristics

Variable	Category	*n* (%)
Overall Cohort	Total beneficiaries	16,990
Sex	Male	9,730 (57.30%)
	Female	7,260 (42.70%)
Age Category	65–74 yo	6,070 (35.70%)
	75–84 yo	6,611 (38.90%)
	85 + yo	4,309 (25.40%)
Race	White beneficiaries	13,439 (79.10%)
	Black beneficiaries	1,438 (8.46%)
	*Other beneficiaries	1,982 (11.70%)
Ethnicity	Hispanic beneficiaries	1,574 (9.26%)
	Non-Hispanic beneficiaries	15,331 (90.20%)
	Unknown Ethnicity beneficiaries	85 (0.50%)
Chronic Conditions	0–1	9,924 (58.40%)
	2–3	3,640 (21.40%)
	3+	3,426 (20.20%)
Income	<= $14,999	3,101 (18.30%)
	$15,000-$39,999	6,423 (37.80%)
	>$40,000	7,466 (43.90%)
Education	High School graduate	3,283 (19.30%)
	Some College	8,284 (48.80%)
	College Degree	4,826 (28.40%)
	Unknown	597 (3.50%)
Income to Poverty Ratio	<=100% of the Federal Poverty Level	2,446 (14.40%)
	> 100% and < = 125% of the Federal Poverty	1,337 (7.90%)
	> 125% and < = 150% of the Federal Poverty	1,250 (7.40%)
	> 150% and < = 200% of the Federal Poverty Level	2,159 (12.70%)
	> 200% of the Federal Poverty Level	9,798 (57.70%)
Primary Predictor: Overall Unmet HRSN	Overall cohort with unmet HRSN (≥ 1 or more)	1,046 (6.20%)
MCBS Unmet HRSN by Question Type		
	Food Insecurity	181 (1.07%)
	Delay in Medical Care due to Cost	560 (3.30%)
	Access to Medical Care	479 (2.82%)
Primary Outcomes	1 ED visit (within 1 year of survey)	4,702 (27.70%)
	Admitted after 1 ED visit	1,535 (9.03%)
	≥ 2 ED visits	2,195 (12.90%)
	Admitted after ≥ 2 ED visits	1081 (6.40%)

*Other Race: (CMS Defined Racial and Ethnic Categories) Hispanic; Asian/Native Hawaiian, or Pacific Islander; American Indian or Alaska Native

### Outcome: 1 ED visit

In univariate analysis, beneficiaries with ≥ 1 unmet HRSN showed no significant difference in odds of 1 ED visit compared to reference (OR 0.99, 95% CI 0.82–1.18) (not included in table). After adjusting for demographic factors, comorbidities, and socioeconomic variables, the association remained non-significant (OR 1.13, 95% CI 0.92–1.38) (Table 2). The fully adjusted model demonstrated that older patients (85 + age category, OR 1.35 CI: 1.14–1.60) and a higher burden of chronic conditions (2–3 conditions: OR 4.40, CI: 3.73–5.19, 4 + conditions: OR 10.09, CI: 8.75–11.64) was associated with an increased odds of ED index visit when compared to reference. Increasing educational attainment was associated with a statistically significant decreased odds of an index ED visit (some college: OR 0.78, CI 0.62–0.99, college degree: OR 0.61, CI: 0.48–0.78). In models incorporating interaction terms, the overall association between HRSN and 1 ED visit remained similar to that in the non-interaction model (OR 1.17, 95% CI 0.72–1.90) (Table 3 and Table S3- S4). However, the magnitude and direction of the relationship varied by age and education. For example, the impact of unmet HRSN decreased with increasing age (age ≥ 85: OR 0.91, 95% CI 0.55–1.51) and among those with a college degree (OR 1.32, 95% CI 0.70–2.32) shifted direction. The adjusted Wald test indicated that the interaction between HRSN and age category (*p* = 0.0112), chronic condition burden (*p* < 0.0001), and education (*p* = 0.0002) was jointly significant. In examining individual interaction terms, Hispanic beneficiaries with unmet HRSN had higher odds of ED utilization compared to non-Hispanic beneficiaries (OR 3.06, 95% CI 1.02–9.18).

**Table 2:** Multivariable model for outcomes of 1 ED visit and within one year of MCBS survey (OR and 95% CI)

Variable	1 ED Visit OR (95% CI)	≥ 2 ED visits OR (95% CI)
**HRSN** ≥ **1 (ref: no HRSN)**	1.13 (0.92–1.38)	1.47 (1.12–1.91)

**Primary predictor shown here**. (see Table S2 for all covariates and full model results). *HRSN *Health related social needs

**Table 3:** Significant HRSN interaction effects for index 1 ED visit and ≥ 2 ED visits. Interaction models test primary predictor (HRSN) with each covariate

Interaction Variable	Odds Ratio	95% CI
**Outcome: 1 ED Visit Interaction Model Terms**		
**HRSN** ≥ **1 (ref: none)**	1.17	0.72–1.90
**Age Category (ref: age 65–74)**		
Age 75–84*	0.89	0.573–1.379
Age 85+*	0.91	0.55–1.51
**Chronic Conditions (ref: 0–1)**		
Chronic Conditions 2–3*	1.04	0.651–1.668
Chronic Conditions 3+*	1.22	0.700–2.121
**Education (ref: high school or less)**		
Education: Some College*	1.03	0.651–1.642
Education: College Degree*	1.32	0.66–2.64
**Beneficiary Ethnicity (ref: non-Hispanic Ethnicity)**		
Hispanic Ethnicity†	3.06	1.02–9.18
**Outcome: ≥2 ED visits Interaction Model Terms**		
**HRSN** ≥ **1(ref: no HRSN)**	1.962	0.96–4.0
**Age Category (ref: age 65–74)**		
Age 75–84*	0.89	0.51–1.55
Age 85+*	1.18	0.65–2.15
**Beneficiary Race (ref: White Race Beneficiaries)**		
Beneficiary Black Race*	1.39	0.48–4.03
Beneficiary Other Race*	1.98	0.61–6.47
**Chronic Conditions (ref: 0–1)**		
Chronic Conditions 2–3*	0.79	0.42–1.47
Chronic Conditions 3+*	0.56	0.31–1.01
**Education (ref: high school or less)**		
Education: Some College*	1.07	0.56–2.04
Education: College Degree*	2.18	0.90–5.30

An * indicates significant joint interaction effect based on adjusted Wald test. † indicates significant individual interaction term (*p* < 0.05) (results of full interaction models in Tables S3- S4)

### Outcome: ≥2 ED visits

Univariate analysis showed that beneficiaries with ≥ 1 unmet HRSN had significantly higher odds of ≥ 2 ED visits (OR 1.23, 95% CI 0.99–1.55) using those without HRSN as reference (not included in table). This association strengthened and remained significant in the fully adjusted model (OR 1.47, 95% CI 1.12–1.91) (Table 2). Other factors significantly associated with increased odds of ≥ 2 ED visits in the adjusted model included older age and higher comorbidity scores, while lower income and education levels were associated with decreased odds. When interaction terms were included, the relationship between HRSN and ED revisits was significantly modified by age, race, chronic conditions, and education. The HRSN effect was higher among beneficiaries aged ≥ 85 (OR 1.18, 95% CI 0.65–2.15) relative to the primary predictor (OR 1.96, 95% CI 0.96–4.0). Racial differences were evident, particularly among Black beneficiaries (OR 1.39, 95% CI 0.48–4.03). The HRSN impact was lowest among those with 3 + chronic conditions (OR 0.56, 95% CI 0.31–1.01). Education had a highly variable effect, with the strongest association among beneficiaries with some college (OR 2.19, 95% CI 0.94–5.10). The adjusted Wald test showed that adding these interactions significantly improved model performance, with age (*p* < 0.001), race (*p* < 0.05), chronic conditions (*p* < 0.001), and education (*p* < 0.001) each demonstrating joint significance in modifying the HRSN–ED revisit relationship.

### Secondary outcomes: hospital admission after 1 ED visit

In univariate analyses, beneficiaries with ≥ 1 unmet HRSN had significantly lower odds of hospital admission (OR 0.61, 95% CI 0.44–0.83) following 1 ED visit (not presented in tables). This association persisted in the fully adjusted model (OR 0.67, 95% CI 0.49–0.92) (Table 3). The adjusted model also showed that male sex, older age, and higher comorbidity scores were associated with increased odds of admission. Significant interactions were found with sex, age, chronic conditions, income-to-poverty ratio, and education by adjusted Wald test. This demonstrated jointly significant interactions, sex (*p* < 0.0001), for age (*p* = 0.0047), for chronic conditions (*p* < 0.0001), and education (*p* = 0.0376) (Table 5).

### Secondary outcomes: hospital admission after ≥ 2 ED visits

Examining hospital admission following the ≥ 2 ED visits outcome in univariate, having one or more unmet HRSN was not significantly associated with hospital admission following ED revisit (OR = 1.03, 95% CI: 0.75–1.41, *p* = 0.870) (not presented in the table). This persisted in the fully adjusted model (OR = 1.21, 95% CI: 0.86–1.68) (Table 4). However, several clinical and demographic factors emerged as significant predictors. Beneficiaries with 4 or more conditions demonstrated a three-fold increase in admission odds (OR = 3.05, 95% CI: 2.27–4.09). Age was also a significant predictor, with beneficiaries aged 85 and older having 75% higher odds of admission compared to those aged 65–74 (OR = 1.75, 95% CI: 1.35–2.27). Educational attainment showed a protective effect, with college graduates having significantly lower odds of admission compared to those with a high school education or less (OR = 0.63, 95% CI: 0.43–0.92). For the interaction model, age and comorbidity levels modified the effect of unmet HRSN on hospital admissions following ED revisits. Of note for this outcome, the presence of multiple chronic conditions attenuated the HRSN effect, with similar reductions seen in both the 2–3 conditions group (OR 0.70, 95% CI 0.20–2.44) and the 3 + conditions group (OR 0.80, 95% CI 0.26–2.43), however also not individually statistically significant. By adjusted Wald test these interactions were jointly statistically significant across age (*p* = 0.0012) and for chronic conditions (*p* < 0.0001) (Table 5).

**Table 4:** Multivariable model for outcomes of hospital admission after 1 ED visit and ≥ 2 ED visits within one year of MCBS survey (OR and 95% CI)

Variable	Admission from 1 ED Visit OR (95% CI)	Admission after ≥ 2 ED visits OR (95% CI)
**HRSN (1 or more)**	0.67 (0.49–0.92)	1.21 (0.86–1.68)

**Primary predictor only shown here** (see supplemental Tables S5 and S6 for all covariates and full model results). *HRSN* Health related social needs, *NA* was not statistically significant

**Table 5:** Multivariable with jointly significant HRSN interaction effects for hospital admission after index 1 ED visit and ≥ 2 ED visits

Interaction Variable	Odds Ratio	95% CI
**Outcome: Admission after 1 ED Visit**		
**Sex (ref: female beneficiaries)**		
Male beneficiaries	1.67	0.83–3.38
**Age Category (ref: 65–74)**		
Age 75–84 yo	0.72	0.32–1.61
Age 85+ yo	0.50	0.17–1.47
**Chronic Conditions (ref: 0–1)**		
Chronic Conditions 2–3	1.67	0.47–5.92
Chronic Conditions 3+	1.36	0.43–4.31
**Education (ref: High School or less)**		
Education Some College	1.87	0.73–4.77
Education College Degree	1.16	0.28–4.77
**Outcome: Admission after ≥ 2 ED visits**		
**Age Category (ref: 65–74)**		
Age 75–84	1.30	0.63–2.66
Age 85+	1.73	0.70–4.29
**Chronic Conditions (ref: 0–1)**		
Chronic Conditions 2–3	0.70	0.20–2.44
Chronic Conditions 3+	0.80	0.26–2.43

Indicates significant joint interaction effect based on adjusted Wald test † indicates significant individual interaction term (p < 0.05) (results of full interaction models in Table S5- S6)

## Discussion

In this study of older adults enrolled in fee-for-service Medicare who completed the MCBS during 2015 − 1016, we found that while the prevalence of self-reported HRSNs were low (6.2% overall for the cohort), they were associated with some significant differences in ED utilization patterns. First, the presence of one or more unmet HRSNs was associated with increased odds of 1 ED visit, although this relationship did not reach statistical significance in the fully adjusted model (OR 1.13, 95% CI 0.92–1.38) Second, and more notably, individuals with unmet HRSNs had significantly higher odds of an ≥ 2 ED visits compared to those without (adjusted OR 1.47, 95% CI 1.12–1.91). Third, somewhat paradoxically, beneficiaries with unmet HRSNs had lower odds of hospital admission following 1 ED visit (adjusted OR 0.67, 95% CI 0.49–0.92), a finding that warrants further investigation. Additionally, our results highlighted the vulnerability of the most elderly population (85 years and older), who demonstrated significantly increased odds of both 1 ED visit and ≥ 2 ED visits compared to younger Medicare beneficiaries. As expected, we also observed that higher comorbidity burden was consistently associated with increased odds of ED utilization across all outcomes. In adjusted Wald tests examining effect modification, we observed that age, education, race/ethnicity, and comorbidity burden each significantly modified the relationship between unmet HRSN and ED utilization—despite many individual interaction terms not reaching statistical significance. Notably, Hispanic ethnicity was the only individually significant interaction term for 1 ED visit, illustrating that the association between unmet HRSN and utilization differs systematically by key demographic factors and clinical characteristics. These findings offer important insights as hospital systems, research institutions, clinicians, and policymakers work to develop strategies to identify and address HRSNs, as well as to better understand their impact on health outcomes. This research also highlights the importance of HRSN screening and structured data collection in the emergency care setting.

These results are consistent with prior literature demonstrating the association between HRSN in Medicare or Medicaid beneficiaries and increased ED utilization [23, 24]. Our findings suggest that ED utilization serves as a critical touchpoint for Medicare beneficiaries with unmet HRSNs. While unmet HRSN burden not associated 1 ED visit, the increased odds of ≥ 2 ED visits point to the persistence of underlying needs may not be fully addressed in outpatient or community settings. Compared to younger patients, older adults utilize emergency services at a higher rate. This increased utilization is associated with deterioration of functional and health status with increasing age and occurs regardless of social needs [25]. We postulate that this may be the reason for a lack of significant increase in index ED visits for individuals with unmet HRSN. Rather than solely interpreting ≥ 2 ED visits as an indicator of gaps in care, they may reflect appropriate access for individuals with acute or ongoing needs. These findings are salient in light of the recent focus on the development of initiatives by CMS to implement widespread screening for HRSNs among all older adults and highlights the importance of addressing HRSNs across the entire Medicare program [26]. In this work we also observed a statistically significant increased odds of 1 ED visit and ≥ 2 ED visits for the most elderly age category (85 years or older) when compared to a reference age category 65–74 years old. Literature featuring data from Medicare beneficiaries and other national databases identified similar findings [27, 28]. The unique needs and characteristics of elderly patients pose significant challenges to the healthcare system. This is of particular importance in the fast-paced environment of the ED where older adults may face concerns of inadequate discharge planning and follow up care, poor medication management and lack of connection to community support services upon discharge that may prove vital especially for individuals with unmet HRSNs [29, 30]. ≥2 ED visits for older patients were also associated with increased morbidity, mortality and healthcare costs [31]. Further work is needed to better identify older adults in the oldest-old age category at high risk of increased healthcare utilization and implement strategies to reduce potential poor outcomes.

Our findings regarding hospital admissions present an interesting contrast to the ED utilization patterns. In both univariate and fully adjusted models, beneficiaries with one or more unmet HRSNs had significantly lower odds of hospital admission following 1 ED visit (adjusted OR 0.67, 95% CI 0.49–0.92). This seemingly paradoxical result warrants careful interpretation. We hypothesize that unmeasured confounding factors may influence admission decisions. For instance, concerns about post-discharge care and follow-up for patients with known social needs might impact admission decisions. It’s also possible that the lower admission rates reflect disparities in care, where patients with unmet HRSNs may not receive needed inpatient care due to systemic biases or communication barriers. Lastly, patients with unmet HRSN may be more likely to use the ED for non-emergent issues compared to those without HRSN, due to limited resources such as access to primary care, medication refills and assistance with navigating services. These ED visits usually result in discharge with the necessary resources or follow up. Previous research has shown a link between higher social risks and fewer non-emergent admissions [32]. Additionally, patients with HRSN experience poorer care coordination [33], which can lead to fewer coordinated admissions for non-emergent conditions with their healthcare team. These findings underscore the complex relationship between social needs and healthcare utilization patterns, highlighting the importance of considering the entire care continuum when addressing HRSNs. Further research is needed to elucidate the mechanisms behind these admission patterns and to ensure that patients with unmet HRSNs are receiving appropriate levels of care across all settings.

Many of the Medicare beneficiaries in our cohort did not have an ED visit during the study period, with only about 7% presenting for 1 ED visit, which is lower than reported in previous literature [34]. Similarly, The low prevalence of unmet HRSN contrasts with other research in this area. Canteburry et al. found nearly half of Medicare Advantage beneficiaries identified at least one unmet need, with food insecurity and financial strain being the most commonly reported needs [23]. We hypothesize that the MCBS sample may represent a healthier subset of Medicare beneficiaries who are more likely to have the resources and time to participate in the survey. However, despite the low overall incidence of ED visits, nearly 50% of those who had an ED index visit returned, and half of these individuals required hospital admission for further care. This indicates that among those utilizing the ED, a significant number faced challenges in maintaining their health once back in the community. Previous studies have suggested potential reasons for this, such as difficulty accessing timely follow-up care, uncertainty about disease progression, and a lack of resources or education to prevent further ED visits and admissions [35]. Further investigation into this Medicare population is necessary to better understand the causal pathways and mechanisms leading to increased ED utilization in the setting of unmet HRSN.

We believe our findings are best interpreted within the context of our proposed conceptual framework—drawing on both the WHO SDOH and Healthy People 2030 [36] —which underscores how the conditions in which people “are born, grow, work, live, and age” fundamentally shape health outcomes. This work also complements Healthy People 2030’s established goals to reduce health disparities. Although no significant statistical association emerged between unmet health-related social needs (HRSNs) and a single ED visit after adjustment, the association was significant for those with ≥ 2 ED visits, suggesting complex and variable utilization patterns meriting further investigation. Consistent with the WHO’s view of health inequities as arising from material deprivation, psychosocial factors, and the biological embedding of social disadvantage, these results may be especially salient for older Medicare beneficiaries whose lifetime exposure to social disadvantage can accumulate over time. Additionally, the paradoxical finding of lower risk of hospital admission among individuals with unmet HRSNs reflects the intricate interplay of access to care, care-seeking behaviors, and clinical decision-making that may impact those with social vulnerabilities. These barriers, highlighted in Healthy People 2030’s Access to Health Services goals, underscore the need for improved access to comprehensive, quality healthcare services—particularly for older adults—to achieve health equity and enhance quality of life.

### Limitations

This study has several limitations. First, we focused solely on a subsample of Medicare beneficiaries who participated in the MCBS during the 2015–2016 calendar year which may not be representative of contemporary trends. Second, the data in the MCBS is derived from patient interviews, and self-reported information is subject to recall bias. Third, because participation in the MCBS interviews is voluntary, our research design may not fully account for selection bias, and there could be unobserved factors influencing the decision to participate or decline in the survey. Lastly, there may be important variables not included in our study or not measured in the MCBS database that could help clarify the relationship between HRSN and ED utilization. Despite these limitations, we believe that utilizing a nationally representative sample of older adults enhances our understanding of the complex relationship between HRSN and healthcare utilization.

## Conclusion

In this study of older adults enrolled in Medicare who participated in the MCBS, self-reported unmet HRSNs had overall low prevalence but were associated with statistically significant increased odds of an ≥ 2 ED visits but not 1 ED visit. Adults in the oldest old age category had statistically significantly higher odds of both 1 ED visit and ≥ 2 ED visits. Approximately 50% of older adults who visited the ED returned within the study period, with half of these individuals requiring admission. These findings highlight the link between HRSNs and ED utilization in older adults as well as the vulnerability of the oldest CMS beneficiaries. Targeted efforts to address HRSNs and better understand the risk factors for ED utilization are essential to meet the needs of this older population.

## Supplementary Information

Supplementary Material 1.

## Data Availability

CMS data for this study is not publicly available but can be requested through standard data use agreements with the Research Data Assistance Center (ResDAC) www.resdac.org.

## Abbreviations

HRSN: Health Related Social Needs
MCBS: Medicare Current Beneficiary Survey
