# Supplementary material for: Are unmet health related social needs associated with emergency department utilization among Medicare beneficiaries?
Source: BMC Health Serv Res. 2025 Mar 31;25:477. doi: 10.1186/s12913-025-12554-7 (PMC11956181; doi:10.1186/s12913-025-12554-7)
Supplement: Supplementary file 1 — Supplementary Material 1. [file 12913_2025_12554_MOESM1_ESM.docx]

Supplemental Tables and Figures

1. Table S1: MCBS HRSN Variables and questions from survey
2. Table S2: Complete multivariable model results for all outcomes OR and 95% Cis (all covariates)
3. Table S3: Full Interactions Model for 1 ED visit
4. Table S4: Full Interactions Model for ≥2 ED visits
5. Table S5: Full Interactions Model for Hospital Admission after 1 ED visit
6. Table S6: Full Interactions Model for Hospital Admission after ≥2 ED visits

**Table S1:** MCBS HRSN Variables and related questions from survey

| **MCBS Survey Domain** | **MCBS Variable Name** | **Survey Question** | **Variable Coding** |
| --- | --- | --- | --- |
| **Primary Predictors** | | | |
| Access to care | HCTROUBL YES1FMT AC29 | Has sample person (SP) had trouble getting needed health care? | Y=1, N=0 |
| Financial Strain | HCDELAY YES1FMT AC31 | Last year ever delay in care due to cost | Y=1, N=0 |
| Food Insecurity | FOODLAST FMTFDLAS FS1 | How often household afford replace food? | Often true/Sometimes true=1  Never true=0 |
| **Covariates** | | | |
| Education | SPDEGRCV DEGREFMT DI3A | Education Level | No HS grad and below = 0  High School graduate = 1  Some College, no degree = 2  Associate’s degree = 3  Bachelor’s degree = 4  Post graduate degree = 5 |
| Financial Strain | IPR_IND IPRIND | Income to Poverty Ratio | 1 <=100% of the Federal Poverty Level  2 >100% and <=125% of the Federal Poverty 3 >125% and <=150% of the Federal Poverty 4 >150% and <=200% of the Federal Poverty 5 >200% of the Federal Poverty Level |

Table S2: Complete multivariable model results for all outcomes OR and 95% CIs

| Variable | 1 ED visit OR (95% CI) | ≥2 ED visits OR (95% CI) | Hospital Admission 1 ED visit OR (95% CI) | Hospital Admission ≥2 ED visits OR (95% CI) |
| --- | --- | --- | --- | --- |
| REF: No HRSN | REF |  |  |  |
| HRSN (1 or more) | 1.13 (0.92-1.38) | 1.47 (1.12-1.91) | 0.67 (0.49-0.92) | 1.21 (0.86-1.68) |
| REF: Age Category 65-74 | 1.00 (1.00-1.00) |  |  |  |
| Age Category: 75-84 | 1.04 (0.90-1.19) | 1.08 (0.90-1.29) | 0.98 (0.79-1.23) | 1.19 (0.94-1.51) |
| Age Category: 85+ | 1.35 (1.14-1.60) | 1.52 (1.28-1.79) | 1.33 (1.06-1.66) | 1.25 (0.94-1.66) |
| REF: Female | REF |  |  |  |
| Sex: Male | 0.91 (0.81-1.02) | 0.95 (0.81-1.11) | 1.49 (1.23-1.79) | 1.75 (1.35-2.27) |
| REF: Race White Beneficiaries | REF |  |  |  |
| Race: Black Beneficiaries | 1.22 (0.92-1.60) | 1.19 (0.89-1.60) | 1.07 (0.76-1.51) | 0.95 (0.59-1.52) |
| Race: Other Beneficiaries | 0.82 (0.59-1.14) | 0.65 (0.43-0.97) | 0.87 (0.46-1.64) | 1.15 (0.52-2.57) |
| Race Unknown: Beneficiaries | 0.83 (0.41-1.70) | 0.35 (0.09-1.33) | 2.22 (0.60-8.21) | 0.77 (0.40-1.48) |
| REF: Ethnicity: Non-Hispanic Beneficiaries | REF |  |  |  |
| Ethnicity: Hispanic: Beneficiaries | 0.98 (0.70-1.39) | 1.21 (0.73-2.01) | 1.12 (0.56-2.22) | 0.56 (0.13-2.43) |
| REF: 0-1 | REF |  |  |  |
| Chronic Conditions: 2-3 | 4.40 (3.73-2.84) | 5.19 (2.52-3.50) | 1.84 (1.46-2.31) | 1.34 (1.00-1.80) |
| Chronic Conditions: 3+ | 10.10 (8.75-6.14) | 11.64 (5.09-7.10) | 3.07 (2.44-3.86) | 3.05 (2.27-4.09) |
| REF: Income: < $14999 | REF |  |  |  |
| Income: $15k-39999 | 1.02 (0.77-1.31) | 1.04 (0.79-1.37) | 0.78 (0.52-1.15) | 0.92 (0.72-1.19) |
| Income: $40000+ | 0.97 (0.73-1.29) | 0.89 (0.62-1.28) | 0.97 (0.61-1.55) | 1.02 (0.70-1.50) |
| REF: Income to Poverty Ratio <100% | REF |  |  |  |
| Income to Poverty Ratio 100-124% | 0.90 (0.71-1.13) | 0.92 (0.69-1.24) | 1.26 (0.89-1.77) | 0.84 (0.58-1.21) |
| Income to Poverty Ratio 125%-149% | 0.77 (0.60-1.07) | 0.87 (0.61-1.25) | 1.85 (1.12-3.06) | 0.71 (0.47-1.07) |
| Income to Poverty Ratio 150%-199% | 0.75 (0.56-1.00) | 0.82 (0.59-1.15) | 1.18 (0.80-1.75) | 0.93 (0.60-1.24) |
| Income to Poverty Ratio >200% | 0.68 (0.52-0.90) | 0.72 (0.52-1.00) | 0.87 (0.57-1.31) | 0.90 (0.63-1.29) |
| REF: Education: HS or less | REF |  |  |  |
| Education: Some College | 0.86 (0.67-1.09) | 0.79 (0.67-0.92) | 0.99 (0.79-1.26) | 0.91 (0.67-1.26) |
| Education: College Degree | 0.68 (0.53-0.88) | 0.60 (0.48-0.75) | 0.73 (0.53-1.01) | 0.63 (0.48-0.75) |
| Education: Unknown | 1.41 (1.00-1.99) | 0.98 (0.70-1.37) | 0.88 (0.61-1.26) | 0.88 (0.64-1.34) |

Table S3: 1 ED visit Full Model with Interactions (interactions in bold)

| Variable | Odds Ratio | Lower CI | Upper CI | P-value |
| --- | --- | --- | --- | --- |
| REF: No HRSN | REF | REF | REF | REF |
| HRSN (1 or more) | 1.035 | 0.501 | 2.138 | 0.925 |
| REF: Female | REF | REF | REF | REF |
| Male | 0.957 | 0.844 | 1.087 | 0.497 |
| **HRSN x male** | **0.686** | **0.399** | **1.179** | **0.17** |
| REF: Age Category 65-74 | REF | REF | REF | REF |
| Age Category: 75-84 | 1.04 | 0.899 | 1.202 | 0.596 |
| Age Category: 85+ | 1.304 | 1.095 | 1.553 | 0.003 |
| **HRSN x Age Category 75-84** | **0.889** | **0.573** | **1.379** | **0.596** |
| **HRSN x Age Category 85+** | **0.913** | **0.551** | **1.511** | **0.72** |
| REF: Race White Beneficiaries | REF | REF | REF | REF |
| Race: Black Beneficiaries | 1.108 | 0.831 | 1.476 | 0.481 |
| Race: Other Beneficiaries | 0.844 | 0.577 | 1.235 | 0.379 |
| Race Unknown: Beneficiaries | 1.009 | 0.474 | 2.15 | 0.98 |
| **HRSN x Race: Black Beneficiaries** | **1.547** | **0.744** | **3.217** | **0.24** |
| **HRSN x Race: Other Beneficiaries** | **0.803** | **0.266** | **2.425** | **0.694** |
| **HRSN x Race Unknown: Beneficiaries** | **0.28** | **0.015** | **5.372** | **0.395** |
| REF: Ethnicity: Non-Hispanic Beneficiaries | REF | REF | REF | REF |
| Hispanic Ethnicity | 0.786 | 0.539 | 1.148 | 0.211 |
| Unknown Ethnicity | 0.87 | 0.375 | 2.019 | 0.744 |
| **HRSN x Hispanic Ethnicity** | **3.061*** | **1.02** | **9.184** | **0.046** |
| **HRSN x Unknown Ethnicity** | **1.855** | **0.228** | **15.064** | **0.56** |
| REF: Chronic Conditions 0-1 | REF | REF | REF | REF |
| Chronic Conditions: 2-3 | 4.44 | 3.729 | 5.287 | 0.0 |
| Chronic Conditions: 3+ | 9.782 | 8.349 | 11.462 | 0.0 |
| **HRSN x Chronic Conditions: 2-3** | **1.042** | **0.651** | **1.668** | **0.863** |
| **HRSN x Chronic Conditions: 3+** | **1.218** | **0.7** | **2.121** | **0.481** |
| REF: Income: < $14999 | REF | REF | REF | REF |
| Income: $15k-39999 | 1.086 | 0.828 | 1.423 | 0.549 |
| Income: $40000+ | 1.075 | 0.793 | 1.456 | 0.639 |
| **HRSN x Income: $15k-39999** | **0.72** | **0.349** | **1.487** | **0.372** |
| **HRSN x Income: $40000+** | **0.787** | **0.31** | **1.994** | **0.61** |
| REF: Income to Poverty Ratio <100% | REF | REF | REF | REF |
| Income to Poverty Ratio 100-124% | 0.961 | 0.739 | 1.249 | 0.763 |
| Income to Poverty Ratio 125%-149% | 0.773 | 0.535 | 1.117 | 0.168 |
| Income to Poverty Ratio 150%-199% | 0.775 | 0.574 | 1.047 | 0.096 |
| Income to Poverty Ratio >200% | 0.68 | 0.512 | 0.903 | 0.008 |
| **HRSN x Income to Poverty Ratio 100-124%** | **0.639** | **0.281** | **1.454** | **0.282** |
| **HRSN x Income to Poverty Ratio 125%-149%** | **1.114** | **0.473** | **2.621** | **0.803** |
| **HRSN x Income to Poverty Ratio 150%-199%** | **0.897** | **0.403** | **1.996** | **0.788** |
| **HRSN x Income to Poverty Ratio >200%** | **1.689** | **0.669** | **4.265** | **0.264** |
| REF: Education: HS or less | REF | REF | REF | REF |
| Education: Some College | 0.852 | 0.741 | 0.981 | 0.026 |
| Education: College Degree | 0.66 | 0.556 | 0.783 | 0.0 |
| Education: Unknown | 1.435 | 1.007 | 2.045 | 0.046 |
| **HRSN x Education: Some College** | **1.033** | **0.651** | **1.642** | **0.888** |
| **HRSN x Education: College Degree** | **1.321** | **0.661** | **2.637** | **0.427** |
| **HRSN x Education: Unknown** | **0.196** | **0.012** | **3.26** | **0.253** |

Table S4: ≥2 ED visits Full Model with Interactions (interaction in bold)

| Variable | Odds Ratio | Lower CI | Upper CI | P-value |
| --- | --- | --- | --- | --- |
| REF: No HRSN | REF | REF | REF | REF |
| HRSN (1 or more) | 1.962 | 0.962 | 4.0 | 0.064 |
| REF: Female | REF | REF | REF | REF |
| Male | 0.994 | 0.836 | 1.182 | 0.945 |
| **HRSN x male** | **0.658** | **0.356** | **1.217** | **0.18** |
| REF: Age Category 65-74 | REF | REF | REF | REF |
| Age Category: 75-84 | 1.101 | 0.912 | 1.329 | 0.315 |
| Age Category: 85+ | 1.512 | 1.269 | 1.803 | 0.0 |
| **HRSN x Age Category 75-84** | **0.892** | **0.513** | **1.553** | **0.684** |
| **HRSN x Age Category 85+** | **1.179** | **0.647** | **2.15** | **0.587** |
| REF: Race White Beneficiaries | REF | REF | REF | REF |
| Race: Black Beneficiaries | 1.133 | 0.847 | 1.515 | 0.398 |
| Race: Other Beneficiaries | 0.581 | 0.366 | 0.923 | 0.022 |
| Race Unknown: Beneficiaries | 0.238 | 0.061 | 0.926 | 0.039 |
| **HRSN x Race: Black Beneficiaries** | **1.393** | **0.481** | **4.034** | **0.538** |
| **HRSN x Race: Other Beneficiaries** | **1.981** | **0.607** | **6.472** | **0.254** |
| **HRSN x Race Unknown: Beneficiaries** | **2.658** | **0.105** | **67.115** | **0.549** |
| REF: Ethnicity: Non-Hispanic Beneficiaries | REF | REF | REF | REF |
| Hispanic Ethnicity | 1.293 | 0.761 | 2.196 | 0.338 |
| Unknown Ethnicity | 3.007 | 1.285 | 7.033 | 0.012 |
| **HRSN x Hispanic Ethnicity** | **0.725** | **0.19** | **2.759** | **0.634** |
| **HRSN x Unknown Ethnicity** | **0.249** | **0.013** | **4.658** | **0.349** |
| REF: Chronic Conditions 0-1 | REF | REF | REF | REF |
| Chronic Conditions: 2-3 | 4.19 | 3.348 | 5.243 | 0.0 |
| Chronic Conditions: 3+ | 10.848 | 8.772 | 13.416 | 0.0 |
| **HRSN x Chronic Conditions: 2-3** | **0.785** | **0.418** | **1.473** | **0.447** |
| **HRSN x Chronic Conditions: 3+** | **0.561** | **0.312** | **1.009** | **0.053** |
| REF: Income: < $14999 | REF | REF | REF | REF |
| Income: $15k-39999 | 1.056 | 0.786 | 1.419 | 0.715 |
| Income: $40000+ | 0.941 | 0.649 | 1.365 | 0.746 |
| HRSN x Income: $15k-39999 | 0.914 | 0.393 | 2.125 | 0.833 |
| HRSN x Income: $40000+ | 0.61 | 0.184 | 2.02 | 0.415 |
| REF: Income to Poverty Ratio <100% | REF | REF | REF | REF |
| Income to Poverty Ratio 100-124% | 1.017 | 0.734 | 1.408 | 0.92 |
| Income to Poverty Ratio 125%-149% | 0.879 | 0.593 | 1.305 | 0.52 |
| Income to Poverty Ratio 150%-199% | 0.885 | 0.63 | 1.244 | 0.479 |
| Income to Poverty Ratio >200% | 0.69 | 0.496 | 0.96 | 0.028 |
| **HRSN x Income to Poverty Ratio 100-124%** | **0.522** | **0.22** | **1.24** | **0.139** |
| **HRSN x Income to Poverty Ratio 125%-149%** | **0.924** | **0.39** | **2.187** | **0.856** |
| **HRSN x Income to Poverty Ratio 150%-199%** | **0.478** | **0.179** | **1.276** | **0.139** |
| **HRSN x Income to Poverty Ratio >200%** | **1.38** | **0.457** | **4.169** | **0.565** |
| REF: Education: HS or less | REF | REF | REF | REF |
| Education: Some College | 0.777 | 0.658 | 0.918 | 0.003 |
| Education: College Degree | 0.543 | 0.439 | 0.672 | 0.0 |
| Education: Unknown | 0.956 | 0.694 | 1.316 | 0.779 |
| **HRSN x Education: Some College** | **1.072** | **0.564** | **2.037** | **0.831** |
| **HRSN x Education: College Degree** | **2.182** | **0.898** | **5.302** | **0.084** |
| **HRSN x Education: Unknown** | **0.267** | **0.019** | **3.832** | **0.328** |

**Table S5: Full Interactions Model for Hospital Admission after 1 ED visit**

| **Variable** | **Odds Ratio** | **Lower CI** | **Upper CI** | **P-value** |
| --- | --- | --- | --- | --- |
| REF: No HRSN | REF | REF | REF | REF |
| HRSN (1 or more) | 0.3719 | 0.1098 | 1.2598 | 0.111 |
| REF: Female | REF | REF | REF | REF |
| Male | 1.4234 | 1.1736 | 1.7264 | 0.0 |
| **HRSN x male** | **1.6735** | **0.829** | **3.3782** | **0.149** |
| REF: Age Category 65-74 | REF | REF | REF | REF |
| Age Category: 75-84 | 1.013 | 0.7932 | 1.2937 | 0.917 |
| Age Category: 85+ | 1.3735 | 1.0863 | 1.7367 | 0.009 |
| **HRSN x Age Category 75-84** | **0.719** | **0.3213** | **1.609** | **0.418** |
| **HRSN x Age Category 85+** | **0.4998** | **0.1696** | **1.4733** | **0.206** |
| REF: Race White Beneficiaries | REF | REF | REF | REF |
| Race: Black Beneficiaries | 1.0575 | 0.7223 | 1.5484 | 0.772 |
| Race: Other Beneficiaries | 0.8487 | 0.4273 | 1.6856 | 0.636 |
| Race Unknown: Beneficiaries | 2.4741 | 0.6007 | 10.1909 | 0.207 |
| **HRSN x Race: Black Beneficiaries** | **1.0055** | **0.223** | **4.5342** | **0.994** |
| **HRSN x Race: Other Beneficiaries** | **1.1692** | **0.1356** | **10.0795** | **0.886** |
| **HRSN x Race Unknown: Beneficiaries** | **NA** | **NA** | **NA** | **NA** |
| REF: Ethnicity: Non-Hispanic Beneficiaries | REF | REF | REF | REF |
| Hispanic Ethnicity | 1.0629 | 0.5116 | 2.2084 | 0.869 |
| Unknown Ethnicity | 0.3566 | 0.0564 | 2.254 | 0.27 |
| **HRSN x Hispanic Ethnicity** | **1.1925** | **0.174** | **8.172** | **0.856** |
| **HRSN x Unknown Ethnicity** | **7.845** | **0.3015** | **204.1189** | **0.213** |
| REF: Chronic Conditions 0-1 | REF | REF | REF | REF |
| Chronic Conditions: 2-3 | 1.261 | 0.9205 | 1.7274 | 0.147 |
| Chronic Conditions: 3+ | 2.0023 | 1.5467 | 2.5921 | 0.0 |
| **HRSN x Chronic Conditions: 2-3** | **1.6719** | **0.472** | **5.9225** | **0.422** |
| **HRSN x Chronic Conditions: 3+** | **1.3625** | **0.4311** | **4.3068** | **0.595** |
| REF: Income: < $14999 | REF | REF | REF | REF |
| Income: $15k-39999 | 0.7271 | 0.477 | 1.1081 | 0.137 |
| Income: $40000+ | 0.8997 | 0.5516 | 1.4673 | 0.669 |
| **HRSN x Income: $15k-39999** | **1.5521** | **0.4572** | **5.2687** | **0.477** |
| **HRSN x Income: $40000+** | **2.5239** | **0.3895** | **16.3548** | **0.328** |
| REF: Income to Poverty Ratio <100% | REF | REF | REF | REF |
| Income to Poverty Ratio 100-124% | 1.3647 | 0.9367 | 1.9883 | 0.104 |
| Income to Poverty Ratio 125%-149% | 2.1088 | 1.214 | 3.6631 | 0.009 |
| Income to Poverty Ratio 150%-199% | 1.2048 | 0.801 | 1.8122 | 0.367 |
| Income to Poverty Ratio >200% | 0.9671 | 0.6304 | 1.4837 | 0.877 |
| **HRSN x Income to Poverty Ratio 100-124%** | **0.5212** | **0.1587** | **1.7122** | **0.28** |
| **HRSN x Income to Poverty Ratio 125%-149%** | **0.3544** | **0.0606** | **2.0742** | **0.247** |
| **HRSN x Income to Poverty Ratio 150%-199%** | **1.1092** | **0.2864** | **4.2956** | **0.88** |
| **HRSN x Income to Poverty Ratio >200%** | **0.3536** | **0.0573** | **2.1835** | **0.26** |
| REF: Education: HS or less | REF | REF | REF | REF |
| Education: Some College | 0.9275 | 0.7196 | 1.1955 | 0.558 |
| Education: College Degree | 0.7016 | 0.5061 | 0.9727 | 0.034 |
| Education: Unknown | 0.8154 | 0.5734 | 1.1596 | 0.253 |
| **HRSN x Education: Some College** | **1.8663** | **0.7308** | **4.7657** | **0.19** |
| **HRSN x Education: College Degree** | **1.156** | **0.2804** | **4.7659** | **0.84** |
| **HRSN x Education: Unknown** | **4.4476** | **0.0571** | **346.2861** | **0.498** |

**Table S6: Full Interactions Model for Hospital Admission after ≥2 ED visits**

| **Variable** | **Odds Ratio** | **Lower CI** | **Upper CI** | **P-value** |
| --- | --- | --- | --- | --- |
| REF: No HRSN | REF | REF | REF | REF |
| HRSN (1 or more) | 0.8269 | 0.2679 | 2.5522 | 0.739 |
| REF: Female | REF | REF | REF | REF |
| Male | 1.1514 | 0.9199 | 1.4413 | 0.216 |
| HRSN x male | **1.637** | **0.7264** | **3.6892** | **0.232** |
| REF: Age Category 65-74 | REF | REF | REF | REF |
| Age Category: 75-84 | 1.2024 | 0.8878 | 1.6286 | 0.231 |
| Age Category: 85+ | 1.6714 | 1.2608 | 2.2158 | 0.0 |
| HRSN x Age Category 75-84 | **1.298** | **0.6334** | **2.6596** | **0.472** |
| HRSN x Age Category 85+ | **1.7278** | **0.6955** | **4.2922** | **0.236** |
| REF: Race White Beneficiaries | REF | REF | REF | REF |
| Race: Black Beneficiaries | 0.9482 | 0.5922 | 1.5182 | 0.823 |
| Race: Other Beneficiaries | 0.66 | 0.3157 | 1.38 | 0.266 |
| Race Unknown: Beneficiaries | 0.6396 | 0.1523 | 2.6854 | 0.538 |
| HRSN x Race: Black Beneficiaries | **1.0445** | **0.2013** | **5.4192** | **0.958** |
| HRSN x Race: Other Beneficiaries | **3.1528** | **0.5089** | **19.5328** | **0.215** |
| HRSN x Race Unknown: Beneficiaries | **None** | **None** | **None** | **None** |
| REF: Ethnicity: Non-Hispanic Beneficiaries | REF | REF | REF | REF |
| Hispanic Ethnicity | 1.4774 | 0.622 | 3.5088 | 0.373 |
| Unknown Ethnicity | 1.866 | 0.5649 | 6.1645 | 0.303 |
| HRSN x Hispanic Ethnicity | **0.1732** | **0.0248** | **1.2119** | **0.077** |
| HRSN x Unknown Ethnicity | **None** | **None** | **None** | **None** |
| REF: Chronic Conditions 0-1 | REF | REF | REF | REF |
| Chronic Conditions: 2-3 | 1.3866 | 1.0082 | 1.9069 | 0.044 |
| Chronic Conditions: 3+ | 3.1528 | 2.2866 | 4.347 | 0.0 |
| HRSN x Chronic Conditions: 2-3 | **0.6949** | **0.1979** | **2.4405** | **0.567** |
| HRSN x Chronic Conditions: 3+ | **0.7981** | **0.2627** | **2.4245** | **0.688** |
| REF: Income: < $14999 | REF | REF | REF | REF |
| Income: $15k-39999 | 0.7908 | 0.5322 | 1.1748 | 0.242 |
| Income: $40000+ | 0.6865 | 0.4487 | 1.0503 | 0.082 |
| HRSN x Income: $15k-39999 | **1.0271** | **0.2966** | **3.5575** | **0.966** |
| HRSN x Income: $40000+ | **0.7412** | **0.0952** | **5.7697** | **0.773** |
| REF: Income to Poverty Ratio <100% | REF | REF | REF | REF |
| Income to Poverty Ratio 100-124% | 0.9035 | 0.6183 | 1.3203 | 0.597 |
| Income to Poverty Ratio 125%-149% | 0.9723 | 0.5624 | 1.681 | 0.919 |
| Income to Poverty Ratio 150%-199% | 0.9676 | 0.6335 | 1.4779 | 0.878 |
| Income to Poverty Ratio >200% | 0.8267 | 0.5612 | 1.2177 | 0.332 |
| HRSN x Income to Poverty Ratio 100-124% | **0.9818** | **0.347** | **2.7777** | **0.972** |
| HRSN x Income to Poverty Ratio 125%-149% | **0.9841** | **0.28** | **3.4583** | **0.98** |
| HRSN x Income to Poverty Ratio 150%-199% | **1.8755** | **0.4471** | **7.8669** | **0.386** |
| HRSN x Income to Poverty Ratio >200% | **2.8219** | **0.7107** | **11.2055** | **0.139** |
| REF: Education: HS or less | REF | REF | REF | REF |
| Education: Some College | 0.9614 | 0.739 | 1.2506 | 0.767 |
| Education: College Degree | 0.6578 | 0.4474 | 0.9671 | 0.033 |
| Education: Unknown | 0.884 | 0.5861 | 1.3332 | 0.553 |
| HRSN x Education: Some College | **0.7508** | **0.3361** | **1.6773** | **0.481** |
| HRSN x Education: College Degree | **0.893** | **0.3277** | **2.4336** | **0.823** |
| HRSN x Education: Unknown | **None** | **None** | **None** | **None** |
